# Folic Acid Supplementation in Pregnancy and the Risk of Pre-Eclampsia—A Cohort Study

**DOI:** 10.1371/journal.pone.0149818

**Published:** 2016-02-22

**Authors:** Shi Wu Wen, Yanfang Guo, Marc Rodger, Ruth Rennicks White, Qiuying Yang, Graeme N. Smith, Sherry L. Perkins, Mark C. Walker

**Affiliations:** 1 OMNI Research Group, Department of Obstetrics and Gynecology, University of Ottawa Faculty of Medicine, Ottawa, Ontario, Canada; 2 Clinical Epidemiology Program, Ottawa Hospital Research Institute, Ottawa, Ontario, Canada; 3 School of Epidemiology, Public Health, and Preventive Medicine, University of Ottawa Faculty of Medicine, Ottawa, Ontario, Canada; 4 Division of Hematology, Department of Medicine, University of Ottawa Faculty of Medicine, Ottawa, Ontario, Canada; 5 Public Health Agency of Canada, Ottawa, Ontario, Canada; 6 Queen’s Perinatal Research Unit, Kingston General Hospital, Kingston, Ontario, Canada; 7 Department of Obstetrics and Gynecology, Queen’s University School of Medicine, Kingston, Ontario, Canada; 8 Division of Biochemistry, The Ottawa Hospital, Ottawa, ON, Canada; VU University Medical Center, NETHERLANDS

## Abstract

This prospective cohort study designed to assess the effect of folic acid supplementation in pregnancy on the risk of preeclampsia (PE) took place in Ottawa, ON and Kingston, ON, Canada, from September 1, 2002 to August 31, 2008. Pregnant women, less than 20 weeks gestational age were recruited and delivered in the Ottawa region and the Kingston General Hospital. Demographic characteristics of the study participants and the patterns of supplementation of folic acid were described and occurrence of PE between women with folic acid supplementation during pregnancy and women without were compared. Multiple logistic regression was used in the estimation of the independent effect of supplementation of folic acid. Additional analyses assessing the effect of low RBC and serum folate and dose-response relationship were performed. Analyses were performed in all study participants, and then in high risk and low risk sub-groups, respectively. A total of 7,669 participants were included in the final analysis. Ninety five percent of the study participants were taking folic acid supplementation in early second trimester. The rate of PE was lower in the supplementation group than in the no supplementation group, and the difference was statistically significant in high risk women. Similar patterns of associations were observed in analysis by RBC and serum folate levels and in dose-response analysis. Folic acid supplementation in pregnancy may reduce PE risk in pregnant women, especially in those women with increased risk of developing PE.

## Introduction

Preeclampsia (PE) is hypertension that develops in pregnancy with proteinuria that affects about 5% of pregnancies [1, 2]. PE is a leading cause of maternal and neonatal morbidity and mortality [3]. In an analysis of the national data of the United States, Zhang et al found that women with PE and eclampsia had a 3- to 25-fold increased risk of abruptio placentae, thrombocytopenia, disseminated intravascular coagulation, pulmonary edema, and aspiration pneumonia, and more than half of women with PE and eclampsia had cesarean delivery [4]. Since delivery is the only known cure, PE is a leading cause of indicated preterm delivery [5]. Means of gestational age were 38.3 and 35.3 weeks (as compared with 39–40 weeks in the general population), and perinatal mortality rates were 2% and 4% (as compared with 5–6 per 1,000 in the general population), respectively, in the mild and severe PE groups [6]. PE accounts for 25% of very low birth weight infants [7] and as many as 60% of these infants suffer from learning disabilities and low IQ [8]. According to our analysis of the Ontario provincial database, the cost of caring for extremely low birth weight infants in the first two years of life attributable to PE in Ontario in 2005 alone could be as high as $19 million [9].

Although the acute endothelial lesion of PE recovers during the postpartum period, women with a history of PE continue to be at risk for future vascular events. Smith et al compared outcomes between women with a history of PE and normotensive women, and suggested that PE increases the cardiovascular risk by 2- to 3-fold one year after childbirth [10]. A Norwegian birth registry identified an 8- to12-fold higher risk of cardiovascular mortality in women with a history of PE and preterm delivery as compared with normotensive women and term delivery [11]. PE may also increase the risk of cardiovascular disease in the offspring through “Developmental Origins of Health and Disease”, or “DOHaD” mechanism [12, 13]. Since no effective treatment/prevention, the impact of PE on both short- and long-term health outcomes in the affected mothers and their offspring remained important. For example, a recent study found that after controlling for potential confounding variables, the risk of maternal PE on very low birth weight (i.e., birth weight < 1,500 g) was increased (1.3, 95% confidence interval 1.2 to 1.5) in infants born between 2003 and 2006 compared with those born between 1994 and 1997 [14].

Previous studies, including our own large cohort studies [15–18], found that folic acid supplementation during pregnancy was associated with a lower incidence of PE. The objective of the proposed study was to further assess the effect of folic acid supplementation in pregnancy on PE risk in a larger birth cohort.

## Methods

Details of the study procedure can be found in our previous publication [15]. In brief, this was a prospective cohort study that recruited pregnant women in Ottawa and Kingston from September 1, 2002 to August 31, 2008 (or the OaK Birth Cohort) at less than 20 weeks gestational age, and who planned to deliver in the Ottawa region and Kingston General Hospital. Patients were initially approached by a clinic nurse at an antenatal visit and were provided information about the purpose of the study. The patients were enrolled into the study after a full explanation of the study purpose and a written consent form has been obtained. The patients had blood drawn for genetic and biochemical analysis. A unique identifier/study number (in the order of entrance to the study) was assigned and was recorded on all clinical and laboratory data. In the meantime, the study nurse collected demographic and life-style data from the participating women by self-report. The delivery and other clinical data were collected within 24–72 hours post-delivery, and a chart review was conducted by the study nurse to collect additional clinical data. Folic acid supplementation (including multivitamins containing folic acid) was obtained from the participating women initially at the time of recruitment and then again at childbirth. If a data element was missing, the research nurses called the participating women within 1 week after childbirth to collect the missing data.

Laboratory investigation was performed in a sub-set of study participants (902 participants in years 2006–2008) to determine the concentrations of red blood cell (RBC) folate. Blood was drawn from the antecubital vein or from the hand of the participating women, and tested using the methods described by Branum et al [19]. Samples were assayed in batches.

PE was defined as a blood pressure greater than 140/90 mmHg on two occasions six hours apart and proteinuria greater than 2+ on dipstick or greater than 300 mg in 24 hour urine collection, supplemented by clinical symptoms and lab test results such as HELLP (hemolysis, elevated liver enzymes, and low platelet count), thrombocytopenia, renal insufficiency, impaired liver function, pulmonary edema, cerebral or visual symptoms, and elevated uric acid levels, in the absence of proteinuria. To ensure the quality of diagnosis of PE, the research coordinator pulled all medical charts identified as PE or gestational hypertension. Adjudication by study investigators (SWW, MR, MW) was conducted to verify the diagnosis for all cases of PE.

We first described the demographic characteristics of the study participants and the patterns of supplementation of folic acid and other vitamins in pregnancy. We then determined the effects of folic acid supplementation on RBC folate levels. Multiple logistic regression was used in the estimation of the independent effect of supplementation of folic acid in early second trimester on PE risk. PE was the dependent variable and folic acid supplementation (yes or no, no as the reference) was the independent variable. Potential confounding variables included in the initial regression models included maternal age (<20, 20–29, 30–34, and > = 35 years, 20–29 as the reference), ethnic background (Caucasians and others, Caucasians as the reference), education (< = 6, 7 to 12, 13–15, and > = 16 years, > = 16 as the reference), parity (first and second or higher births, second or higher as the reference), previous health problems (chronic hypertension, history of preeclampsia, and diabetes, yes or no, no as the reference), pre-pregnancy body mass index (in quartile, the third quartile as reference), gestational age at recruitment, and cigarette smoking (yes and no, no as the reference). Full model with all potential confounding variables included was used in all analyses. These factors were known risk factors of PE and were also related to folic acid supplementation, although the associations may not necessarily be statistically significant in the current study sample. The effects of low RBC folate on PE risk were examined. Low RBC folate was defined as <10^th^ percentile of each gestational week at which the blood was taken. Additional analysis assessing the dose-response relationship was performed. Doses of folic acid were calculated by the brand name of supplementation that the pregnant women were taking in early second trimester (for women with supplementation of two or more types of vitamins, folic acid contained in all vitamins was calculated) and was grouped into the following categories: 0 (no supplementation), 0.1–0.9 mg, 1.0 mg, 1.1–1.9 mg, 2.0–4.0 mg, and >4.0 mg. Analyses of the association between folic acid and PE were performed first in all study participants, and then in high risk and low risk sub-groups, respectively. High risk pregnancy was defined as the presence of any of the following conditions: pre-pregnancy body mass index > = 35, PE in a previous pregnancy, chronic hypertension, diabetes, and multiple pregnancy. Supplement analyses using serum folate and comparing the effect of folic acid on the risk of PE between women who initiated supplementation before or after conception were also conducted. All statistical analyses were performed using Statistical Analysis System, Version 9.1 (SAS Institute Inc., Cary, North Carolina, USA).

## Results and Discussion

A total of 13,238 women were approached to participate in the study and 8,085 (61%) agreed and were recruited into the study. Among them, 416 women were excluded because of missing information such as gestational age at delivery, birth weight or sex (234) and lost to follow up due to participant’s relocation outside of study centre (182), leaving 7,669 participants for final analysis.

Ninety five percent of the study participants were taking folic acid supplementation in early second trimester; most by taking multivitamins containing folic acid at a dose of 1.0 mg or higher. The same pattern of supplementation was observed in high risk and low risk women (Table 1).

**Table 1:** Patterns of folic acid supplementation.

Categories of supplementation	Frequency	Percent (%)
**Overall participants**		
No supplementation	404	5.27
Prenatal vitamin	5313	69.28
Regular Multivitamins	175	2.28
Folic acid alone	625	8.15
Two or more type of vitamins	1152	15.02
**Dose of folic acid (mg)** *		
0	404	5.32
0.1–0.9	241	3.17
1	5834	76.82
1.1–1.9	318	4.19
2.0–4.0	673	8.86
>4.0	124	1.63
High risk pregnant women**		
No supplementation	56	6.36
Prenatal vitamin	564	64.09
Regular Multivitamins	16	1.82
Folic acid alone	74	8.41
Two or more type of vitamins	170	19.32
**Low risk of pregnant women**		
No supplementation	348	5.13
Prenatal vitamin	4749	69.95
Regular Multivitamins	159	2.34
Folic acid alone	551	8.12
Two or more type of vitamins	982	14.46

*75 cases missing information on folic acid dose

** High risk of pregnancy: body mass index > = 35, previous preeclampsia history, chronic hypertension, diabetes, multiple pregnancy.

The majority of the study participants were Caucasian with high socioeconomic status. Women with no supplementation were more likely to be younger, multiparous, non-Caucasians, having a lower education level and household income, and whom smoked cigarettes during pregnancy, than women with supplementation (Table 2).

**Table 2:** Demographic and clinical characteristics of the pregnant women with and without folate supplementation who participated in the Oak Birth cohort study.

Variables	No supplementation (%)	Supplementation (%)	Total	Value	P
	N = 404	N = 7265			
**Maternal age (y)**					
<20	24 5.94	150 2.06	174	36.48	< .0001
20–29	184 45.54	2850 39.23	3034		
30–34	120 29.70	2721 37.45	2841		
> = 35	76 18.81	1544 21.25	1620		
**Maternal background**					
Aboriginal	0 0.00	38 0.52	38	29.11	< .0001
White	280 69.31	5441 74.89	5721		
Middle Eastern	12 2.97	171 2.35	183		
Africa	15 3.71	76 1.05	91		
Asian	19 4.70	342 4.71	361		
Other	78 19.31	1197 16.48	1275		
**Maternal background**					
Causasians	358 88.61	6638 91.37	6996	3.63	0.06
Other	46 11.39	627 8.63	673		
**Prepregnancy body mass index (kg/m**^**2**^**)**					
<18.5	33 8.17	388 5.34	421	19.59	0.001
18.5–24	193 47.77	4160 57.26	4353		
15–29	97 24.01	1644 22.63	1741		
> = 30	47 11.63	661 9.10	708		
> = 35	34 8.42	412 5.67	446		
**Education level**					
high school and below	122 30.27	999 13.76	1121	106.07	< .0001
college/university not completed	60 14.89	696 9.59	756		
college/university completed	221 54.84	5564 76.65	5785		
**Household income ($, CAD)**					
<25000	62 15.78	354 4.95	416	129.20	< .0001
25,000–49,999	97 24.68	1094 15.29	1191		
50,000–79,999	104 26.46	1986 27.75	2090		
> = 80,000	111 28.24	3374 47.14	3485		
**Parity**					
0	125 30.94	3725 51.27	3850	63.29	<0.001
> = 1	279 69.06	3540 48.73	3819		
**Smoking during pregnancy**					
Yes	80 19.80	793 10.92	873	29.96	< .0001
No	324 80.20	6472 89.08	6796		
**Alcohol use**					
Yes	4 0.99	55 0.76	59	0.27	0.60
No	400 99.01	7206 99.24	7606		
**Chronic hypertension**					
Yes	7 1.75	86 1.19	93	0.98	0.32
No	392 98.25	7120 98.81	7512		
**Type1 diabetes**					
Yes	3 0.75	57 0.79	60	0.01	0.93
No	395 99.25	7147 99.21	7542		
**Type2 diabetes**					
Yes	3 0.75	57 0.79	60	0.01	0.93
No	395 99.25	7146 99.21	7541		
**Previous preeclampsia**					
Yes	16 3.96	214 2.95	230	1.35	0.25
No	388 96.04	7044 97.05	7432		
**Gestational age at recruitment (wks)**					
< = 12	218 53.96	4241 58.38	4459	3.16	0.21
13–15	118 29.21	1950 26.84	2068		
16–20	68 16.83	1074 14.78	1142		

Folic acid supplementation was associated with increased RBC folate (Table 3). The association between supplementation and RBC folate was stronger in blood samples drawn at later gestations (Table 3).

**Table 3:** RBC folate levels in participants with folic acid supplementation versus those without, by gestational age at recruitment OaK Birth Cohort Study, October 2002 to December 2008.

Variables	No Supplementation	Supplementation	P-value
RBC folate level(Mean ± SD, nmol/L)*	1311.0±399.3	1610.3±441.1	0.0002
RBC folate level stratified by gestational weeks at recruitment (Mean ± SD, nmol/L)			
≤ 12	1270.8±459.0	1587.7±427.9	0.0037
13–15	1322.0±381.4	1634.8±458.0	0.0211
16–20	1439.0±173.0	1776.2±487.2	0.1793

* 902 participants have the information on RBC folate level

The rate of PE was lower in the supplementation group (either supplementation with multiple vitamins containing folic acid or folic acid alone) than in the no supplementation group, and the difference was statistically significant in women with increased risk of PE (Table 4). On the other hand, no significant association between low RBC folate levels with risk of PE was found (Table 4).

**Table 4:** Adjusted odds ratio (OR) and 95% confidence interval (95%CI) of preeclampsia.

Variables	Number of participants	Preeclampsia (%)	ORs (95% CI)*
**All women**			
Folic acid supplementation			
No	404	17 (4.21)	Reference
Yes	7265	228 (3.14)	0.58 (0.33, 1.02)
Folic acid supplementation alone			
No	404	17 (4.21)	Reference
Yes	625	24 (3.84)	0.76 (0.36, 1.62)
RBC folate			
>10^th^ percentile	766	19 (2.48)	Reference
< = 10^th^ percentile	92	3 (3.26)	1.99 (0.53, 7.38)
Women with high risk pregnancy**			
Folic acid supplementation			
No	56	8 (14.29)	Reference
Yes	824	76 (9.22)	0.42 (0.18, 0.98)
Folic acid supplementation alone			
No	56	8 (14.29)	Reference
Yes	74	6 (8.11)	0.17 (0.03, 0.95)
RBC folate			
>10^th^ percentile	94	5 (5.32)	Reference
< = 10^th^ percentile	6	1 (16.67)	4.60 (0.29, 73.013)
**Women with low risk pregnancy**			
Folic acid supplementation			
No	348	9 (2.59)	Reference
Yes	6441	152 (2.36)	0.75 (0.34, 1.63)
Folic acid supplementation alone			
No	348	9 (2.59)	Reference
Yes	551	18 (3.27)	1.35 (0.52, 3.51)
RBC folate			
>10^th^ percentile	672	14 (2.08)	Reference
< = 10^th^ percentile	86	2 (2.33)	1.55 (0.31, 7.64)

*Adjusted for maternal age, previous health problem (chronic hypertension, history of preeclampsia, diabetes), smoking, and parity.

** High risk of pregnancy: body mass index > = 35, previous preeclampsia history, chronic hypertension, diabetes, multiple pregnancy.

No statistically significant dose-response relationship between folic acid supplementation and the risk of PE (P > 0.05 for trend test) was found (Table 5).

**Table 5:** Occurrences of PE according to folate dose groups.

Dose group (mg)	Number of participants	Number of Preeclampsia	Rate of PE (%)	χ2	P value*
**All cases**					
0	404	17	4.21		
0.1–0.9	241	6	2.49		
1	5834	175	3.00	7.81	0.32
1.1–1.9	318	13	4.09		
2.0–4.0	673	24	3.57		
> = 4.0	124	8	6.45		
**Women with high risk pregnancy**					
0	56	8	14.29		
0.1–0.9	24	2	8.33		
1	629	55	8.74	0.37	0.98
1.1–1.9	42	5	11.90		
2.0–4.0	92	13	14.13		
>4.0	26	1	3.85		
**Women with low risk pregnancy**					
0	348	9	2.59		
0.1–0.9	217	4	1.84		
1	5205	120	2.31	11.00	0.37
1.1–1.9	276	8	2.90		
2.0–4.0	581	11	1.89		
>4.0	98	7	7.14		

* P value for Cochran-Armitage trend test

Similar patterns were found for women with different serum folate concentrations and no difference in the effect of folic acid on the risk of PE between women who initiated supplementation before or after conception was found (Table 6).

**Table 6:** Association between serum folate concentration and PE and according time of folic acid supplementation.

	n of subjects	n of PE cases	%	P
**Serum folate**				> 0.05
>10^th^ percentile	6,803	211	3.10	
< = 10^th^ percentile	747	28	3.75	
**Initiation time of supplementation**				
Before conception	3,034	96	3.16	> 0.05
After conception	4,018	127	3.16	

### Main findings

Our prospective study in a cohort of Canadian women found that about 95% had supplementation with folic acid or multivitamins containing folic acid in early second trimester, and among them, most had a supplementation of 1.0 mg or higher. Supplementation of multivitamins containing folic acid was associated with a lower rate of PE, and the results were statistically significant in women with increased risk of developing PE. Although some of the differences were not statistically significant, the patterns in point estimates in different analyses were quite consistent: by overall supplementation, by RBC folate concentrations, in overall study participants, or in participants with high risk pregnancy and participants with low risk pregnancy.

### Strengths and limitations

Our study was designed to test a biologically plausible hypothesis in a large prospective cohort of women, with fairly detailed information on folic acid supplementation and clinical data, allowing refined analysis. All cases of PE were adjudicated by study investigators so errors in ascertainment of outcome were reduced. In addition to supplementation data, we measured RBC and serum folate levels.

A major limitation of our study is that the non-supplementation was rare (5%) during the study period. Such a low non-supplementation rate could result in major selection bias and confounding difficult to be controlled for in observational studies. RBC folate level was measured only in a selected subset of participants (902 participants in years 2006–2008). Although overall study sample was fairly large, the power of the study was limited because the number of non-supplementation was small and the event rate was low. Because of the high supplementation rate, only 3.35% of the study subjects were folate insufficient according to the World Health Organization standard for women in reproductive age (folate concentration < 15.9 nmol/L) [20]. We calculated the powers for three set of analysis and they are all indeed low: the power for folate supplementation was only 25%, for RBC folate level it was 10%, and for serum folate level it was 18%. The issue of limited sample size is severe in important sub-analyses as the sample diminished rapidly because of the need to stratify by risk status and by doses in these analyses.

### Interpretation

The finding of significant reduction in PE risk associated with folic acid supplementation in women with increased risk of developing PE was consistent with our Phase I of OaK Birth Cohort study which included 2,951 participants recruited from The Ottawa Hospital and Kingston General Hospital between October 2002 and December 2005, which found a statistically significant lower rate of PE in the supplementation group than in the no supplementation group (aOR of 0.37 and 95% CI of 0.18, 0.75) [15]. Several earlier studies by other groups also found that folic acid supplementation reduced the risk of PE [17, 18]. In a large population based historical cohort study, we found that the risks of PE (adjusted odds ratio (OR) 1.52, 95% confidence interval (95% CI): 1.39, 1.66) and severe PE (OR: 1.77, 95% CI: 1.38, 2.28) were increased in mothers with folic acid antagonists exposure [16]. Folic acid antagonists include a broad spectrum of drugs with a common mechanism of depleting maternal folate. Findings from the effect of maternal exposure to folic acid antagonists on the increased risk of PE added to the weight of evidence that folic acid supplementation may decrease the risk of PE. Moreover, folic acid supplementation was found to be associated with several other adverse pregnancy outcomes such as placental abruption [21, 22], fetal loss [21], and fetal growth restriction [21, 23] that are considered to share the same mechanism of placental pathology by Ray and Laskin [24]. Nilsen et al analyzed data from 280,127 singletons collected by Medical Birth Registry of Norway and found that compared with no folic acid supplementation, any supplementation was associated with a 26% risk reduction of placental abruption (adjusted OR = 0.74, 95% CI 0.65, 0.84) [25]. Plasma or serum homocysteine is strongly associated with folate intake, with increased homocysteine levels associated with lower folate intake. Vollset et al analyzed the association between plasma homocysteine and PE, and found that the adjusted risk for PE was 32% higher in women with upper quartile of plasma homocysteine level as compared with women with lower quartile [26]. On the other hand, a recent large cohort study involving 5,593 pregnant women, which showed no effect of folic acid on PE or gestational hypertension [27]. There are a couple of reasons that may explain the discrepant results from different studies. First, in recent studies in industrialized countries, non-supplementation became very rare. In our Phase I OaK Birth Cohort study, about 8% women had no supplementation of folic acid (15). In Phase 2 of the Oak Birth Cohort (the current cohort), which was the expansion of the Phase I OaK Birth Cohort, 5,000 additional participants were recruited and added to Phase 1, and the overall non-supplementation rate dropped to 5%. The non-supplementation rate in the Timmermans study was also about 5% [27]. When the rate of non-supplementation became rare, the reasons for non-supplementation may be highly selective. As a result, selection bias/confounding may become difficult to detect and control. Second, the association between folic acid supplementation and PE may depend on the risk profile of the study population. Couple of studies found that folic acid supplementation can lower the risk of PE only in lean women [17, 28]. We speculate that this may be caused by the dose problem, as in both the Bodnar’s [17] and Catov’s [28] studies, most women had supplementation of 0.4 mg per day. In our cohort, most women had supplementation of 1 mg per day. Because of the potential genetic and metabolic defects, women with increased risk may need a higher dose [29–31]. In our study, we tested the hypothesis that a high dose of folic acid supplementation may be needed for women with increased risk of PE by analyzing data for a subset sample of women with pre-pregnancy body mass index > = 35, previous PE history, chronic hypertension, diabetes, or multiple pregnancy. The results of analyses in women with increased risk of developing PE were statistically significant, may be because high dose folic acid is truly beneficial to these women.

## Conclusion

In this large prospective cohort study, we found that folic acid supplementation in pregnancy was associated with reduced risk of PE, although the association was statistically significant only in women with increased risk of developing PE. Large scale randomized controlled trials to definitively prove or disprove the effect of folic acid supplementation in pregnancy on PE, are urgently needed.
